# Clinical performance of DNA‐based prenatal screening using single‐nucleotide polymorphisms approach in Thai women with singleton pregnancy

**DOI:** 10.1002/mgg3.1256

**Published:** 2020-04-24

**Authors:** Tachjaree Panchalee, Naravat Poungvarin, Warisa Amornrit, Julaporn Pooliam, Pattarawalai Taluengjit, Tuangsit Wataganara

**Affiliations:** ^1^ Department of Obstetrics and Gynecology Mahidol University Bangkok Thailand; ^2^ Department of Clinical Pathology Mahidol University Bangkok Thailand; ^3^ Research Department Mahidol University Bangkok Thailand; ^4^ Division of Clinical Epidemiology Faculty of Medicine Siriraj Hospital Mahidol University Bangkok Thailand

**Keywords:** autosomal trisomy, DNA‐based screening, Down syndrome, noninvasive prenatal screening, sex chromosomal aneuploidies, single nucleotide polymorphisms

## Abstract

**Background:**

To review the performance of noninvasive prenatal screening (NIPS) using targeted single‐nucleotide polymorphisms (SNPs) approach in mixed‐risk Thai women.

**Methods:**

Retrospective analysis of data for detection of trisomy 21 (T21), 18 (T18), 13 (T13), monosomy X (XO), other sex chromosome aneuploidies (SCA), and triploidy/vanishing twins (VT) from a single commercial laboratory.

**Results:**

Mean (±*SD*) gestational age and maternal weight were 13.2 (±2.1) weeks and 125.7 (±22.4) pounds, respectively (*n* = 8,572). From 462/8,572 (5.4%) no‐calls; 1/462 (0.2%) was uninformative SNPs, and 1/462 chose amniocentesis. Redraw settled 323/460 (70%) samples with low fetal fraction (FF); and 8,434/8,572 (98.4%) were finally reportable, with 131 high risks (1.6%). The median (min‐max) FF of reportable (*n* = 8,434) and unreportable samples (*n* = 137) samples were 10.5% (2.6–37.9) and 3.8% (1–14.1), respectively (*p* < .05). Fetal karyotypes were available in 106/131 (80.9%) and 52/138 (37.7%) high risk and repeated no‐calls, respectively. The positive predictive values (PPVs) for T21 (*n* = 47), T18 (*n* = 15), T13 (*n* = 7), XO (*n* = 8), other SCA (*n* = 7), and triploidy/VT were 94%, 100%, 58.3%, 66.7%, 70%, and 57.1%, respectively. None of repeated no‐calls had aneuploidies.

**Conclusion:**

SNP‐based NIPS has high PPVs for T21 and T18. Although the proprietary SNPs library is not population‐specific, uninformative SNPs are uncommon.

## Graphical abstract

Single‐nucleotide polymorphism (SNP)‐based noninvasive prenatal screening has high positive predictive values for trisomy 21 and 18. Although the proprietary SNPs library is not population‐specific, uninformative SNPs are uncommon.

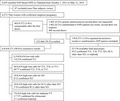

## 1 BACKGROUND

Down syndrome (trisomy 21; T21) imposes a great burden to the society. The prevalence of T21 in Thailand is 1.2 per 1,000 live births (Jaruratanasirikul et al., 2017). High‐throughput DNA‐based noninvasive prenatal screening (NIPS) has been rapidly adopted in clinical cares since it became commercially available in late 2011. Recent large‐scale studies have consistently reported excellent performance of NIPS to detect fetal T21, trisomy 18 (T18), trisomy 13 (T13), and sex chromosome aneuploidies (SCA) in the first and second trimester of pregnancy (Bianchi et al., 2014; Cuckle, Benn, & Pergament, 2015; Gil et al., 2017; Hui et al., 2015; McCullough et al., 2014; Nicolaides, Syngelaki, Ashoor, Birdir, & Touzet, 2012; Sago, Sekizawa, & Japan, 2015; Samura et al., 2017; Shaw, Chen, & Cheng, 2013; Willems et al., 2014; Zhang et al., 2015). Most NIPS platforms utilize quantitative “counting” methods (whole‐genome sequencing; WGS) where fetal chromosome copy number is determined by comparing the absolute number of sequence reads from the chromosome(s) of interest (ie chromosome 21) to reference chromosome(s). Fetal trisomy is inferred when this ratio is above a predetermined threshold (Futch et al., 2013). Fetal fraction (FF), the amount of the cell‐free DNA in the maternal blood that is of fetal origin, is essential for accurate test results (Wataganara, Bui, Choy, & Leung, 2016). Placental production (apoptosis) and renal excretion of cell‐free DNA may be varied in different racial origins (DiNonno et al., 2019; Heazell, Whitworth, Duley, & Thornton, 2015; Ryan et al., 2016; Wataganara, Chen, et al., 2005; Wataganara, Metzenbauer, Peter, Johnson, & Bianchi, 2005b). However, previous publications suggested that performance of WGS‐based NIPS is not affected by ethnic backgrounds (Bianchi et al., 2012; Manotaya et al., 2016).

The NIPS using WGS and single‐nucleotide polymorphism (SNP) approaches may perform differently (Cuckle, 2017; Salomon et al., 2017). Unique advantages of SNP‐based NIPS are (a) differentiation between maternal and fetal contributions of the sequence reads, thus help flagging the samples with maternal mosaicism which may cause false positive (FP) results, and (b) detection of additional haplotypes, thus help identifying triploidy, uniparental disomy (molar pregnancy), and vanishing twin (VT) which may escape WGS methods (Levy & Norwitz, 2013). If needed, additional sets of SNPs are targeted for identification of microdeletions (Gross et al., 2016). However, SNPs approach may have a higher no‐call rate than WGS methods, especially in women with consanguinity, surrogacy, and transplantation of solid organ or bone marrow (Zimmermann et al., 2012). A clinical performance study of SNP‐based NIPS performed in USA reported approximately 80% positive predictive value (PPV) with very low negative predictive value (NPV) (Dar et al., 2014). Published data from SNP‐based NIPS are more limited than those of WGS approach (Badeau et al., 2017). The proprietary library, which is not population‐specific, contained 11,000 to 19,488 SNPs covering chromosomes 21, 18, 13, X, and Y to determine allele identity (Zimmermann et al., 2012). The library has recently been truncated to 13,392 SNPs (Ryan et al., 2016). We aimed to assess our population‐specific performance of SNPs‐based NIPS due to the concerns of possible differences of SNP allele frequency and FF in Thai women.

## 2 METHODS

### 2.1 Ethical compliance

This study was approved by the Siriraj Institutional Review Board (COA Si 742/2017) as parts of an assessment prior to technology transfer with our academic laboratory (Department of Clinical Pathology, Faculty of Medicine Siriraj Hospital).

This is a retrospective analysis of collected data from SNP‐based NIPS (Panorama^TM^, Natera Inc.) in Thailand from October 1, 2013 until May 31, 2018. At the time of data collection, the test was exclusively self‐paid. Only samples from pregnant women with singleton pregnancy were included. Samples were excluded in cases of gestational age <9 weeks, multiple gestation, donor egg pregnancy, surrogate carrier, missing patient information or incomplete consent documents, sample received >6 days after collection, insufficient blood volume (<13 ml), wrong collection tube used, or if the sample was apparently damaged. This study was conducted prior to the twins panel of this test has become available (Norwitz et al., 2019).

Written informed consent was obtained from all women opted for SNP‐based NIPS. Maternal age (calculated from date of birth), weight, and gestational age were routinely requested for each sample. Samples that passed quality‐control metrics were processed at a single commercial laboratory (Natera Inc.). Validated methodologies were used for isolation of cell‐free DNA, polymerase chain reaction amplification of targeting SNPs on chromosomes 21, 18, 13, X, and Y, high‐throughput sequencing, and risk scoring with a proprietary algorithm (Nicolaides, Syngelaki, Gil, Atanasova, & Markova, 2013; Pergament et al., 2014). The FF was estimated from allelic ratios of SNPs on chromosomes that are never trisomic or monosomic in a viable pregnancy, and was embedded in the SNP sequencing process (Wataganara et al., 2016). All samples with a risk score ≥99/100 and <1/10,000 were reported as high‐ and low‐risk for aneuploidies, respectively. Additional haplotypes detected on SNPs sequencing heightened the risks of triploidy/VT. A second blood draw (redraw) was requested if total cell‐free DNA, FF, or signal‐to‐noise ratio failed quality‐control metrics. This could be due to sample impurity, insufficient yield of DNA after extraction, or failure of DNA sequencing. Redraw was not offered in cases of uninformative SNPs pattern including; large regions (>25%) loss of heterozygosity, poor fit of the data to the model, or suspected maternal or fetal mosaicism (Dar et al., 2014). Genetic amniocentesis was offered to those with high‐risk calls or repeated inconclusive results (no‐calls).

Follow‐up information on high‐risk results was obtained by a single partner laboratory. (Bangkok Cytogenetics Center Co. Ltd.) Clinical follow‐up was completed in December 2018. Results were categorized as follows: (a) true positive (TP: high‐risk samples that were confirmed by prenatal or postnatal diagnostic testing, or based on clinical evaluation at birth), (b) false positive (FP: high‐risk samples that were shown to be euploid by follow‐up testing or based on clinical evaluation at birth, (c) false negative (FN: low‐risk samples that were reported as aneuploid by the providers). To encourage more validated positive cases and to find FPs, an insurance policy was provided by the partner laboratory from May 2017 to reimburse the cost of invasive diagnostic tests for women with high‐risk call. This insurance policy could also minimize unreported FNs because providers were motivated to report of missed calls forT21, T18, and T13, whereby the woman would be reimbursed for 100,000 Thai Bahts (approximately 3,000 US Dollars).

### 2.2 Statistical analysis

Categorical variables were expressed as number and percentage. Continuous variables were expressed as percentage, means, standard deviation (*SD*), median and ranges (minimum‐maximum). Samples with conclusive and repeated inconclusive results (no‐calls) were compared for maternal age, weight, gestational age at first draw, and FF, using independent *t* test or Mann–Whitney *U* test. Due to wide distribution of FF and relatively small sample size, FFs were not expressed as gestational week‐specific multiples of the median, and linear regression analysis between FF and other demographic variables were not performed (Ashoor, Syngelaki, Poon, Rezende, & Nicolaides, 2013). The performance was defined by aneuploidy‐specific PPV (TP/(TP + FP)).

Because most of the low‐risk samples were not confirmed by genetic test at the time of birth, true negative (TN) could not be accurate. We therefore chose not to calculate NPV (TN/(TN + FN)). Sensitivity and specificity were not reported because our screened population was biased (not every Thai women received SNP‐based NIPS).

SPSS version 18 (SPSS Inc.) was used for analyzing data. P value less than 0.05 was considered significant.

## 3 RESULTS

**FIGURE 1:**
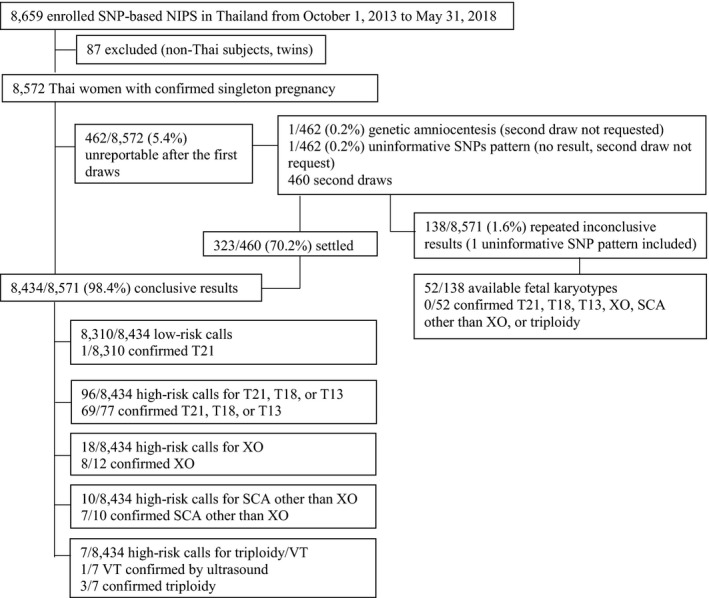
Flow chart for results of the study. NIPS, noninvasive prenatal screening; SCA, sex chromosome aneuploidies; SNP, single nucleotide polymorphism; T13, trisomy 13; T18, trisomy 18; T21, trisomy 21; VT, vanishing twin; XO, monosomy X

The study enrollment (*n* = 8,659) is summarized in Figure 1. The majority of the samples were from private medical providers. (Bangkok Cytogenetics Center Co. Ltd., personal communication) After exclusion of 87 samples from non‐Thai women and twins, 8,572 samples were analyzed. Indications for NIPS are as follows; maternal age ≥35 years (*n* = 3,874; 45.2%), parental anxiety (physician discretion) (*n* = 2,897:33.8%), positive first trimester combined test (>1:250) (*n* = 1,569:18.3%), abnormal ultrasound examination (*n* = 189:2.2%), and undefined (*n* = 43:0.5%). Comparative analysis of the test performance in each category was not performed due to inadequacy of power. After the first draw, the tests were unreportable in 462 women (5.4%), of which 1 (0.2%) was due to uninformative SNP pattern. One woman opted for genetic amniocentesis at this point. For 460 women with low FF, a second blood draw could settle 323 (70%) cases. At the end, the tests were conclusive in 8,434 samples (98.4%); with 131 high‐risk calls (1.6%). After exclusion of 1 woman who immediately opted for genetic amniocentesis, and inclusion of 1 uninformative SNPs pattern, there were138 women (1.6%) with repeated inconclusive results.

Baseline characteristics are summarized in Table 1. Maternal age and gestational age of samples with conclusive and repeated inconclusive results were not significantly different (*p* > .05). Women with reportable results had significantly lower weight and higher FF than those with repeated inconclusive results (*n* = 136; after exclusion of 2 subjects; 1 who chose genetic amniocentesis after low FF from the first draw and 1 with uninformative SNPs pattern) (*p* < .01).

**TABLE 1:** Characteristics of samples with conclusive and repeated inconclusive results

	Total (*n* = 8,571^a^)	Conclusive results (*n* = 8,434)	Repeated inconclusive results (*n* = 137)	*p* value
MA (years) (mean ± *SD*)	35.0 ± 3.5	35 ± 3.5	35.1 ± 4.4	.6
MW (lbs.) (mean ± *SD*)	125.7 ± 22.4	125.2 ± 22.0	134.7 ± 26.7	**<.001**
GA at first draw; mean ± *SD*	13.2 ± 2.1	13.2 ± 2.1	13.3 ± 2.1	.3
FF; median (%) (min‐max)	N/A^b^	10.5% (2.6–37.9)	3.8% (1–14.1)^c^ (*n* = 80)	**<.001**

Abbreviations: FF, fetal fraction; GA, gestational age; lbs, pounds; MA, maternal age; MW, maternal weight; *SD*, standard deviation; SNP, single‐nucleotide polymorphism.

^a^ After exclusion of one woman who opted for genetic amniocentesis after FF in the first draw was too low. One uninformative SNP pattern was included in repeated inconclusive result.

^b^ Median FF could not be calculated because 57/137 (42%) of repeated inconclusive results had unmeasurable FF.

^c^ Median (min‐max) was calculated from those with measurable FF (80/137), yet unreportable result.

Table 2 demonstrates the clinical performance of SNP‐based NIPS. Results of confirmatory genetic test were available in of 106/131 (80.9%) of those with high‐risk calls. The PPVs for T21, T18, and T13 were 94%, 100%, and 58.3%, respectively, with an average PPV for detection of these 3 common autosomal trisomies of 89.6%. The PPVs for monosomy X (XO), SCA other than XO, and triploidy/VT were 66.7%, 70%, and 57.1%, respectively. Fetal karyotypes were available in 52/138 women with repeated unreportable results (1 uninformative SNP included), none of which had aneuploidies. We did not have molar pregnancy reported in this cohort.

**TABLE 2:** Clinical performance of single‐nucleotide polymorphism‐based noninvasive prenatal screening in Thai women with conclusive results (*n* = 8,434)

Types of Aneuploidy	High‐risk calls	Confirmatory testing (%)	TP	FP	PPV (%) (95% CI)	FN
T21	63	50 (79.4)	47	3	94 (83.5–98.0)	1
T18	20	15 (75)	15	0	100.0	0
T13	13	12 (92.3)	7	5	58.3 (36.8–77.1)	0
Overall (T21 + T18+T13)	96	77 (80.2)	69	8	89.6 (81.2–94.5)	0
XO	18	12 (66.7)	8	4	66.7 (42.9–84.2)	0
SCA other than XO	10	10 (100)	7	3	70 (50.5–81.8)	0
Triploidy/VT	7	7 (100)	4	3	57.1 (44.9–87.9)	0
Total	131	106 (80.9)	84	15	84.9 (77.2–90.3)	0

Abbreviations: CI, confidence interval; FP, false positive; PPV, positive predictive value; SCA, sex chromosome aneuploidies; T13, trisomy 13; T18, trisomy 18; T21, trisomy 21; TP, true positive; VT, vanishing twin; XO, monosomy X.

Table 3 compares the performance of SNP‐based NIPS from previous publications searchable in Pubmed. Searching terms were single nucleotide polymorphism, NIPS, noninvasive prenatal testing, Panorama. Systematic reviews were excluded. The PPVs for detection of T21 and T18 in Thai women were high, and comparable with other previous publications.

**TABLE 3:** Summary of performance of single nucleotide‐based noninvasive prenatal screening for detection of trisomy 21 (T21), trisomy 18 (T18), trisomy 13 (T13), monosomy X (XO), sex chromosome aneuploidies (SCA) other than XO, and triploidy

Publication	Sample size	Ethnicity	Study design^a^	Priori risk	GA (weeks)	FF (%)	No call (%)	T21 PPV (%)	T18 PPV (%)	T13 PPV (%)	XO PPV (%)	SCA PPV (%)	Triploidy PPV (%)	Remarks
Zimmermann B, et al. Prenat Diagn 2012	166	N/A	Prospective	Women with known fetal karyotypes	Median: 17 (euploid samples) and 17.5 (aneuploidies samples)	Mean 12 (2.0–30.8)	0	100 (11/11)	100% (3/3)	100 (2/2)	100 (1/1)	66.7 (2/3)	N/A	Development study
Nicolaides KH, et al. Prenat Diagn 2013	242	N/A	External validation	Women underwent CVS for fetal karyotyping	Median 13.1 (11.3–13.9)	N/A	5.4 (13/242)	100 (25/25)	100 (3/3)	100 (1/1)	100 (2/2)	100 (1/1)	100 (1/1)	External validation study
Samango‐Sprouse C, et al. Prenat Diagn 2013	201	N/A	Cross‐sectional	Archived samples with known fetal karyotype	Mean 13.2 (euploid samples) and 15.3 (aneuploid samples)	Mean 10.9 (euploid samples) and 12.1 (aneuploid samples)	6 (12/201)	N/A	N/A	N/A	N/A (sensitivity 91.7% (CI: 61.5–99.8)	100 (3/3)	N/A	Development study for SCA
Pergament E, et al. Obstet Gynecol 2014	1,064	N/A	Prospective	Mixed risk	Median 14.1 (7.6–40.6: euploid samples) and 14.6 (8–38.9; aneuploid samples)		8.1 (85/1,064)	98.1 (103/105)	100 (58/58)	12/12 (100)	90 (9/10)	N/A	N/A	Clinical experience study
Dar P, et al. AJOG 2014	30,705	N/A	Retrospective	Mixed‐risk	Median 12.6 (3.1–40.9)	Mean 10.2 (GA‐specific *SD* available in the paper)	1 (317/30,705)	90.9 (140/154)	93.1 (27/29)	38.1 (8/21)	50 (9/18)	N/A	N/A	Large‐scale clinical experience study
Hall MP, et al. PLoS One 2014	64	N/A	Case‐control	Archived samples with known fetal karyotype	Median 16 (12.1–22.7)	Median 11.1 (2.2–30.4)	*N*/A	*N*/A	*N*/A	100 (15/15)	*N*/A	*N*/A	*N*/A	Development study for T13
Nicolaides KH, et al. Fetal Diagn Ther 2014	56	Caucasian 82.1% (46/56), Afro‐Caribbean 10.7% (6/56), Asian 7.2% (4/56)	Case‐control	Archived samples with known fetal karyotype	11–13	Median 10.1 (3.5–18.1: euploid samples) 23.4 (14.3–40.8: diandric triploid samples), 2.8 (1.4–3.5: digynic triploid samples)	8.3 (4/48 of euploid samples)	N/A	N/A	N/A	N/A	N/A	100 (?)(4/4 detection of multiple paternal haplotypes, suggesting either diandric triploidy or dizygotic twins)	Study for triploidy
Curnow KJ, et al. AJOG 2015	30,795	N/A	Retrospective	Mixed	Median 12.6 (3.1–40.9)	Mean 10.2 (GA‐specific *SD* available in the paper)	1 (317/30,705)	N/A	N/A	N/A	N/A	N/A	96.1 (?) (73/76 confirmed diandric triploidy and twins)	Large‐scale clinical experience study for triploidy, molar, and vanishing twins from detection of additional fetal haplotypes
Eiben B, et al. Ultrasound Int Open 2015	2,942	N/A	Retrospective	Mixed	>9	Mean 10.2 (GA‐specific *SD* available in the paper)	2.2 (66/2,942)	97.4 (38/39)	88.9 (8)	62.5 (5/8)	80 (4/5)	N/A	100 (4/4)	Clinical experience in Germany and Austria
Ryan A, et al. FDT 2016	587	N/A	Case‐control	Archived samples with known fetal karyotype	Median 13 (9.0–36.7; euploid samples) and 14.6 (9.0–36.0: aneuploid samples)	Median 10 (2.0–46.6 euploid samples) and 11.6 (1.4–50.0 aneuploid samples)	2.3	N/A (sensitivity 99.4% (166/167)), specificity 100% (373/373))	N/A (sensitivity 100% (28/28), specificity 100% (512/512))	N/A (sensitivity 100% (14/14), specificity 100% (526/526))	N/A (sensitivity 100% (7/7), specificity 100% (533/533)	N/A	N/A (sensitivity 100% (4/4), specificity 100% (540/540)	Validation of enhanced test version
DiNonno W, et al. J Clin Med 2019	1,035,844	N/A	Retrospective	Mixed	N/A	N/A	N/A	95.7 (1,036/1,083)	93.9 (447/476)	79.6 (148/186)	91.0 (272/299)	N/A	N/A	Only for available follow‐up information for pregnancies in women with high‐risk SNP‐based NIPS results from 2014–2017
Panchalee T, et al.	8,572	Thai 100% (8,572/8,572)	Retrospective	Mixed	Median 10.5 (2.6–37.9)	Mean 13.2 ± 2.1	1.6% (138/8,571) (1 uninformative SNP pattern included)	94 (47/50)	100 (15/15)	58.3 (7/12)	66.7 (8/12)	70 (7/10)	57.1 (4/7)	Clinical experience in Thailand

Abbreviations: CVS, chorionic villous sampling; FF, fetal fraction; GA, gestational age; N, No; N/A, not available; NIPS, noninvasive prenatal screening; PPV, positive predictive value; SCA, sex chromosome aneuploidies other than XO; *SD*, standard deviation; SNP, single‐nucleotide polymorphism; Y, Yes.

^a^ Prospective = samples drawn prior to invasive prenatal diagnosis, archived samples = drawn as part of existing screening program with known outcomes, mixed = mixed of both study design.

## 4 DISCUSSION

High PPVs for detection of T21 and T18 in Thai women of mixed baseline risks with SNP‐based NIPS are consistent with studies conducted elsewhere (Table 3). The PPVs are prevalence‐dependent, and can be used for counseling in specific population. Our redraw rate of 5.4% was similar to a previous study (redraw 5.4% at ≥10 weeks’ gestation) (Dar et al., 2014). Repeated inconclusive results (no calls) in Thai population are relatively low (1.6%); and persistently low FF remains an important cause. Although the SNPs library of the test was not developed from the population intended to screen, uninformative SNPs pattern is not a common reason for no‐calls. Our sample size was too small for calculation of PPVs fortriploidy and VT.

Approximately 79% of women in our cohort chose to have SNP‐based NIPS as a first‐tier test due to advanced age and maternal anxiety (physician discretion). Public health care system in Thailand does not subsidize first trimester screening for T21, and combined first trimester screening or NIPS are self‐paid. Thai women need to choose between combined tests and NIPS; considering the advantages and limitations of different technologies, specific gestational situations, psychological status, and financial constraints. Prenatal diagnosis of common aneuploidies in Thailand is often made by genetic amniocentesis in the second trimester. This makes the landscape of prenatal screening and diagnosis in Thailand quite different from developed countries whose data dominate the publication domain.

Although most of human genomes are identical, a relatively small number of SNP, whichis an individual variation at a single position in DNA sequence that occurs in about every 300nucleotides,are varied in different ancestral descents (Yang, Wang, Lin, Chen, & Chen, 2012; Zhou & Wang, 2007). Instrumental SNP markers for a genealogic study are chosen according to their ethnic‐specific distribution (Tocharoentanaphol et al., 2008). The selection of <20,000 SNPs on chromosome of interest from 10 million SNPs in human genome to create the proprietary genomiclibrary was confidential (Wataganara et al., 2016). Algorithm of SNP‐based NIPS is morecomplex than those of WGS approaches, as it tends to a very shallow depth of sequencing at any given polymorphic locus. Measurement of FF with SNPs approach is equally accurate across populations because the quantitation assays are unbiased, uniformly covering chromosome 1 to 12, and maximizing the number of informative loci by targeting SNPs with high minor‐allele frequencies in the HapMap dataset (ftp://ftp.ncbi.nlm.nih.gov/hapmap/) (Juneau et al., 2014; Schmid et al., 2018; Sparks, Struble, Wang, Song, & Oliphant, 2012). Sequencing of SNPs may be the most accurate method to estimate FF because foreign SNPs (paternal/fetal derived) are readily apparent in the woman's plasma (Wataganara et al., 2016). Persistently low FF remains our most important cause of no‐call and FN (Barrett et al., 2017; Canick, Palomaki, Kloza, Lambert‐Messerlian, & Haddow, 2013; Kim et al., 2015). Low FF has been linked with high maternal weight or body mass index (Wataganara, Peter, Messerlian, Borgatta, & Bianchi, 2004). Because FF is highly dynamic, re‐draw could settle only 70% of the cases with low FF from the first draw (Ashoor et al., 2013).

The main limitation of our real‐life clinical performance study was the incomplete post‐test follow‐ups, particularly on low‐risk patients, thus precluding precise calculation of sensitivity, specificity, and NPV (Dar et al., 2014). Some high‐risk calls may not have received confirmatory genetic testing due to the following reasons; (a) spontaneous fetal losses soon after NIPS, (b) unreported elective termination of pregnancy without karyotype confirmation, and (c) unreported birth of newborn babies with T21 due to parental concern of social stigma. Selections of subjects in most of the NIPS studies, including ours, were biased (Badeau et al., 2017). The SNP‐based NIPS should not replace first trimester scan because the test is not always informative, and the PPVs for T13, XO, and SCA is relatively low. Dedicated first‐trimester anomaly scan can identify about 95% of fetuses with T18, T13, triploidy, and Turner syndrome (Wagner, Sonek, Hoopmann, Abele, & Kagan, 2016). Progressive improvement of bioinformatics algorithm is likely to reduce redraw, missed call, and no‐call for those with very low FF (Larson et al., 2018; McKanna et al., 2019).

## CONFLICT OF INTEREST

Faculty of Medicine Siriraj Hospital had technology transfer agreement with Natera Inc., USA and Bangkok Cytogenetics Center Co. Ltd., Thailand. Neither of them was involved with analysis of data and preparation of the manuscript. T.P., N.P, and T.W. have received travel bursary from Bangkok Cytogenetics Ltd. and Natera Inc. to actively participate in their sponsored lecture events. The other authors declare no conflicts of interest. We want to thank Alex Ngo (Natera Inc.) and Arnond Lertphiboon (Bangkok Cytogenetics Center Co. Ltd.) for technical support of this paper. We also want to thank Suparat Jaingam, Chutima Yaiyiam, and Supitchaya Surasereewong for their administrative assistance.

## AUTHOR CONTRIBUTIONS

Tachjaree Panchalee contributed to data gathering and analysis. Naravat Poungvarin contributed to data gathering and analysis. Warisa Amornrit contributed to data gathering and analysis. Julaporn Pooliam contributed to statistical analysis. Pattarawalai Taluengjit contributed to data gathering and analysis. Tuangsit Wataganara contributed to overall supervision.

## Data Availability

The data that support the findings of this study are available from the corresponding author upon reasonable request.

## References

[mgg31256-bib-0001] Ashoor, G. , Syngelaki, A. , Poon, L. C. , Rezende, J. C. , & Nicolaides, K. H. (2013). Fetal fraction in maternal plasma cell‐free DNA at 11–13 weeks' gestation: Relation to maternal and fetal characteristics. Ultrasound in Obstetrics and Gynecology, 41, 26–32. 10.1002/uog.12331 23108725

[mgg31256-bib-0002] Badeau, M. , Lindsay, C. , Blais, J. , Nshimyumukiza, L. , Takwoingi, Y. , Langlois, S. , … Rousseau, F. (2017). Genomics‐based non‐invasive prenatal testing for detection of fetal chromosomal aneuploidy in pregnant women. Cochrane Database Systematic Review, 11, CD011767. 10.1002/14651858.CD011767.pub2 PMC6486016 29125628

[mgg31256-bib-0003] Barrett, A. N. , Xiong, L. , Tan, T. Z. , Advani, H. V. , Hua, R. , Laureano‐Asibal, C. , … Choolani, M. (2017). Measurement of fetal fraction in cell‐free DNA from maternal plasma using a panel of insertion/deletion polymorphisms. PLoS ONE, 12, e0186771 10.1371/journal.pone.0186771 29084245 PMC5662091

[mgg31256-bib-0004] Bianchi, D. W. , Parker, R. L. , Wentworth, J. , Madankumar, R. , Saffer, C. , Das, A. F. , … … GROUP, C. S. (2014). DNA sequencing versus standard prenatal aneuploidy screening. New England Journal of Medicine, 370, 799–808. 24571752 10.1056/NEJMoa1311037

[mgg31256-bib-0005] Bianchi, D. W. , Platt, L. D. , Goldberg, J. D. , Abuhamad, A. Z. , Sehnert, A. J. , & Rava, R. P. ; Maternal, B. I. S. S. T. A. D. F. A. S. G. (2012). Genome‐wide fetal aneuploidy detection by maternal plasma DNA sequencing. Obstetrics and Gynecology, 119, 890–901. 10.1097/aog.0b013e31824fb482 22362253

[mgg31256-bib-0006] Canick, J. A. , Palomaki, G. E. , Kloza, E. M. , Lambert‐Messerlian, G. M. , & Haddow, J. E. (2013). The impact of maternal plasma DNA fetal fraction on next generation sequencing tests for common fetal aneuploidies. Prenatal Diagnosis, 33, 667–674. 10.1002/pd.4126 23592541

[mgg31256-bib-0007] Cuckle, H. (2017). cfDNA screening performance: Accounting for and reducing test failures. Ultrasound in Obstetrics and Gynecology, 49, 689–692. 10.1002/uog.17492 28429561

[mgg31256-bib-0008] Cuckle, H. , Benn, P. , & Pergament, E. (2015). Cell‐free DNA screening for fetal aneuploidy as a clinical service. Clinical Biochemistry, 48, 932–941. 10.1016/j.clinbiochem.2015.02.011 25732593

[mgg31256-bib-0009] Curnow, K. J. , Wilkins‐Haug, L. , Ryan, A. , Kırkızlar, E. , Stosic, M. , Hall, M. P. , … Gross, S. J. (2015). Detection of triploid, molar, and vanishing twin pregnancies by a single‐nucleotide polymorphism‐based noninvasive prenatal test. American Journal of Obstetrics and Gynecology, 212, 79.e1–79.e9. 10.1016/j.ajog.2014.10.012 25447960

[mgg31256-bib-0010] Dar, P. , Curnow, K. J. , Gross, S. J. , Hall, M. P. , Stosic, M. , Demko, Z. , … Benn, P. (2014). Clinical experience and follow‐up with large scale single‐nucleotide polymorphism‐based noninvasive prenatal aneuploidy testing. American Journal of Obstetrics and Gynecology, 211, 527.e1–527.e17. 10.1016/j.ajog.2014.08.006 25111587

[mgg31256-bib-0011] Dinonno, W. , Demko, Z. , Martin, K. , Billings, P. , Egbert, M. , Zneimer, S. , … Benn, P. (2019). Quality assurance of non‐invasive prenatal screening (NIPS) for fetal aneuploidy using positive predictive values as outcome measures. Journal of Clinical Medicine, 8, 1311 10.3390/jcm8091311 PMC6780279 31454954

[mgg31256-bib-0012] Eiben, B. , Krapp, M. , Borth, H. , Kutur, N. , Kreiselmaier, P. , Glaubitz, R. , … Merz, E. (2015). Single nucleotide polymorphism‐based analysis of cell‐free fetal DNA in 3000 cases from Germany and Austria. Ultrasound International Open, 1, E8–E11. 10.1055/s-0035-1555765 27689149 PMC5023199

[mgg31256-bib-0013] Futch, T. , Spinosa, J. , Bhatt, S. , de Feo, E. , Rava, R. P. , & Sehnert, A. J. (2013). Initial clinical laboratory experience in noninvasive prenatal testing for fetal aneuploidy from maternal plasma DNA samples. Prenatal Diagnosis, 33, 569–574. 10.1002/pd.4123 23592485 PMC3709117

[mgg31256-bib-0014] Gil, M. M. , Brik, M. , Casanova, C. , Martin‐Alonso, R. , Verdejo, M. , Ramirez, E. , & Santacruz, B. (2017). Screening for trisomies 21 and 18 in a Spanish public hospital: From the combined test to the cell‐free DNA test. J Matern Fetal Neonatal Med, 30, 2476–2482. 10.1080/14767058.2016.1253062 27806655

[mgg31256-bib-0015] Gross, S. J. , Stosic, M. , McDonald‐Mcginn, D. M. , Bassett, A. S. , Norvez, A. , Dhamankar, R. , … Benn, P. (2016). Clinical experience with single‐nucleotide polymorphism‐based non‐invasive prenatal screening for 22q11.2 deletion syndrome. Ultrasound in Obstetrics and Gynecology, 47, 177–183. 10.1002/uog.15754 26396068 PMC5064640

[mgg31256-bib-0016] Hall, M. P. , Hill, M. , Zimmermann, B. , Sigurjonsson, S. , Westemeyer, M. , Saucier, J. , … Rabinowitz, M. (2014). Non‐invasive prenatal detection of trisomy 13 using a single nucleotide polymorphism‐ and informatics‐based approach. PLoS ONE, 9, e96677 10.1371/journal.pone.0096677 24805989 PMC4013011

[mgg31256-bib-0017] Heazell, A. E. P. , Whitworth, M. , Duley, L. , & Thornton, J. G. (2015). Use of biochemical tests of placental function for improving pregnancy outcome. Cochrane Database of Systematic Reviews, CD011202 10.1002/14651858.cd011202.pub2 26602956 PMC8860184

[mgg31256-bib-0018] Hui, L. , Teoh, M. , Da Silva Costa, F. , Ramsay, P. , Palma‐Dias, R. , & Richmond, Z. , … … Australian, N. C. (2015). Clinical implementation of cell‐free DNA‐based aneuploidy screening: Perspectives from a national audit. Ultrasound in Obstetrics and Gynecology, 45, 10–15. 10.1002/uog.14699 25323392

[mgg31256-bib-0019] Jaruratanasirikul, S. , Kor‐Anantakul, O. , Chowvichian, M. , Limpitikul, W. , Dissaneevate, P. , Intharasangkanawin, N. , … Sriplung, H. (2017). A population‐based study of prevalence of Down syndrome in Southern Thailand. World J Pediatr, 13, 63–69. 10.1007/s12519-016-0071-5 27878784

[mgg31256-bib-0020] Juneau, K. , Bogard, P. E. , Huang, S. , Mohseni, M. , Wang, E. T. , Ryvkin, P. , … Zahn, J. M. (2014). Microarray‐based cell‐free DNA analysis improves noninvasive prenatal testing. Fetal Diagnosis and Therapy, 36, 282–286. 10.1159/000367626 25228026

[mgg31256-bib-0021] Kim, S. K. , Hannum, G. , Geis, J. , Tynan, J. , Hogg, G. , Zhao, C. , … Deciu, C. (2015). Determination of fetal DNA fraction from the plasma of pregnant women using sequence read counts. Prenatal Diagnosis, 35, 810–815. 10.1002/pd.4615 25967380

[mgg31256-bib-0022] Larson, N. B. , Wang, C. , Na, J. , Rowsey, R. A. , Highsmith, W. E. , Hoppman, N. L. , … Klee, E. W. (2018). Improving single‐nucleotide polymorphism‐based fetal fraction estimation of maternal plasma circulating cell‐free DNA using Bayesian hierarchical models. Journal of Computational Biology, 25, 1040–1049. 10.1089/cmb.2018.0056 29932737

[mgg31256-bib-0023] Levy, B. , & Norwitz, E. (2013). Non‐invasive prenatal aneuploidy testing: technologies and clinical implication. MLO Medical Laboratory Observer, 45, 8, 10, 12 passim; quiz 16. 23875437

[mgg31256-bib-0024] Manotaya, S. , Xu, H. , Uerpairojkit, B. , Chen, F. , Charoenvidhya, D. , Liu, H. , … Jiang, H. (2016). Clinical experience from Thailand: Noninvasive prenatal testing as screening tests for trisomies 21, 18 and 13 in 4736 pregnancies. Prenatal Diagnosis, 36, 224–231. 10.1002/pd.4775 26748603

[mgg31256-bib-0025] McCullough, R. M. , Almasri, E. A. , Guan, X. , Geis, J. A. , Hicks, S. C. , Mazloom, A. R. , … Saldivar, J. S. (2014). Non‐invasive prenatal chromosomal aneuploidy testing–clinical experience: 100,000 clinical samples. PLoS ONE, 9, e109173 10.1371/journal.pone.0109173 25289665 PMC4188614

[mgg31256-bib-0026] McKanna, T. , Ryan, A. , Krinshpun, S. , Kareht, S. , Marchand, K. , Grabarits, C. , … Benn, P. (2019). Fetal fraction‐based risk algorithm for non‐invasive prenatal testing: Screening for trisomies 13 and 18 and triploidy in women with low cell‐free fetal DNA. Ultrasound in Obstetrics and Gynecology, 53, 73–79. 10.1002/uog.19176 30014528 PMC6587793

[mgg31256-bib-0027] Nicolaides, K. H. , Syngelaki, A. , Ashoor, G. , Birdir, C. , & Touzet, G. (2012). Noninvasive prenatal testing for fetal trisomies in a routinely screened first‐trimester population. American Journal of Obstetrics and Gynecology, 207, 374.e1–374.e6. 23107079 10.1016/j.ajog.2012.08.033

[mgg31256-bib-0028] Nicolaides, K. H. , Syngelaki, A. , del Mar, G. M. , Quezada, M. S. , & Zinevich, Y. (2014). Prenatal detection of fetal triploidy from cell‐free DNA testing in maternal blood. Fetal Diagnosis and Therapy, 35, 212–217. 10.1159/000355655 24135152

[mgg31256-bib-0029] Nicolaides, K. H. , Syngelaki, A. , Gil, M. , Atanasova, V. , & Markova, D. (2013). Validation of targeted sequencing of single‐nucleotide polymorphisms for non‐invasive prenatal detection of aneuploidy of chromosomes 13, 18, 21, X, and Y. Prenatal Diagnosis, 33, 575–579. 10.1002/pd.4103 23613152

[mgg31256-bib-0030] Norwitz, E. R. , McNeill, G. , Kalyan, A. , Rivers, E. , Ahmed, E. , Meng, L. , … Hedriana, H. L. (2019). Validation of a single‐nucleotide polymorphism‐based non‐invasive prenatal test in twin gestations: Determination of zygosity, individual fetal sex, and fetal aneuploidy. Journal of Clinical Medicine, 8, 937 10.3390/jcm8070937 PMC6679081 31261782

[mgg31256-bib-0031] Pergament, E. , Cuckle, H. , Zimmermann, B. , Banjevic, M. , Sigurjonsson, S. , Ryan, A. , … Rabinowitz, M. (2014). Single‐nucleotide polymorphism‐based noninvasive prenatal screening in a high‐risk and low‐risk cohort. Obstetrics and Gynecology, 124, 210–218. 10.1097/aog.0000000000000363 25004354 PMC4144440

[mgg31256-bib-0032] Ryan, A. , Hunkapiller, N. , Banjevic, M. , Vankayalapati, N. , Fong, N. , Jinnett, K. N. , … Hill, M. (2016). Validation of an enhanced version of a single‐nucleotide polymorphism‐based noninvasive prenatal test for detection of fetal aneuploidies. Fetal Diagnosis and Therapy, 40, 219–223. 10.1159/000442931 27028530

[mgg31256-bib-0033] Sago, H. , Sekizawa, A. , & Japan, N. C. (2015). Nationwide demonstration project of next‐generation sequencing of cell‐free DNA in maternal plasma in Japan: 1‐year experience. Prenatal Diagnosis, 35, 331–336. 10.1002/pd.4539 25408438

[mgg31256-bib-0034] Salomon, L. J. , Alfirevic, Z. , Audibert, F. , Kagan, K. O. , Paladini, D. , Yeo, G. , … Committee, I. C. S. (2017). ISUOG updated consensus statement on the impact of cfDNA aneuploidy testing on screening policies and prenatal ultrasound practice. Ultrasound in Obstetrics and Gynecology, 49, 815–816. 10.1002/uog.17483 28573775

[mgg31256-bib-0035] Samango‐Sprouse, C. , Banjevic, M. , Ryan, A. , Ryan, A. , Sigurjonsson, S. , & Zimmermann, B. … Rabinowitz, M. (2013). SNP‐based non‐invasive prenatal testing detects sex chromosome aneuploidies with high accuracy. Prenatal Diagnosis, 33, 643–649. 10.1002/pd.4159 23712453 PMC3764608

[mgg31256-bib-0036] Samura, O. , Sekizawa, A. , Suzumori, N. , Sasaki, A. , Wada, S. , Hamanoue, H. , … Sago, H. (2017). Current status of non‐invasive prenatal testing in Japan. Journal of Obstetrics and Gynaecology Research, 43, 1245–1255. 28586143 10.1111/jog.13373

[mgg31256-bib-0037] Schmid, M. , White, K. , Stokowski, R. , Miller, D. , Bogard, P. E. , Valmeekam, V. , & Wang, E. (2018). Accuracy and reproducibility of fetal‐fraction measurement using relative quantitation at polymorphic loci with microarray. Ultrasound in Obstetrics and Gynecology, 51, 813–817. 10.1002/uog.19036 29484786 PMC6001636

[mgg31256-bib-0038] Shaw, S. W. , Chen, C. P. , & Cheng, P. J. (2013). From Down syndrome screening to noninvasive prenatal testing: 20 years' experience in Taiwan. Taiwanese Journal of Obstetrics and Gynecology , 52, 470–474. 10.1016/j.tjog.2013.10.003 24411028

[mgg31256-bib-0039] Sparks, A. B. , Struble, C. A. , Wang, E. T. , Song, K. , & Oliphant, A. (2012). Noninvasive prenatal detection and selective analysis of cell‐free DNA obtained from maternal blood: Evaluation for trisomy 21 and trisomy 18. American Journal of Obstetrics and Gynecology, 206, 319.e1–319.e9. 10.1016/j.ajog.2012.01.030 22464072

[mgg31256-bib-0040] Tocharoentanaphol, C. , Promso, S. , Zelenika, D. , Lowhnoo, T. , Tongsima, S. , Sura, T. , … Sakuntabhai, A. (2008). Evaluation of resequencing on number of tag SNPs of 13 atherosclerosis‐related genes in Thai population. Journal of Human Genetics, 53, 74–86. 10.1007/s10038-007-0220-8 18043865

[mgg31256-bib-0041] Wagner, P. , Sonek, J. , Hoopmann, M. , Abele, H. , & Kagan, K. O. (2016). First‐trimester screening for trisomies 18 and 13, triploidy and Turner syndrome by detailed early anomaly scan. Ultrasound in Obstetrics and Gynecology, 48, 446–451. 10.1002/uog.15829 26611869

[mgg31256-bib-0042] Wataganara, T. , Bui, T. H. , Choy, K. W. , & Leung, T. Y. (2016). Debates on fetal fraction measurement and DNA‐based noninvasive prenatal screening: Time for standardisation? BJOG, 123(Suppl 3), 31–35. 10.1111/1471-0528.14197 27627594

[mgg31256-bib-0043] Wataganara, T. , Chen, A. Y. , Leshane, E. S. , Sullivan, L. M. , Borgatta, L. , Bianchi, D. W. , & Johnson, K. L. (2005). Changes of cell‐free fetal DNA in maternal plasma after elective termination of pregnancy. Clinical Chemistry, 51, 217–219. 10.1373/clinchem.2004.042135 15528293

[mgg31256-bib-0044] Wataganara, T. , Metzenbauer, M. , Peter, I. , Johnson, K. L. , & Bianchi, D. W. (2005). Placental volume, as measured by 3‐dimensional sonography and levels of maternal plasma cell‐free fetal DNA. American Journal of Obstetrics and Gynecology, 193, 496–500. 10.1016/j.ajog.2004.12.015 16098876

[mgg31256-bib-0045] Wataganara, T. , Peter, I. , Messerlian, G. M. , Borgatta, L. , & Bianchi, D. W. (2004). Inverse correlation between maternal weight and second trimester circulating cell‐free fetal DNA levels. Obstetrics and Gynecology, 104, 545–550. 10.1097/01.aog.0000137352.93110.15 15339767

[mgg31256-bib-0046] Willems, P. J. , Dierickx, H. , Vandenakker, E. , Bekedam, D. , Segers, N. , Deboulle, K. , & Vereecken, A. (2014). The first 3,000 non‐invasive prenatal tests (NIPT) with the Harmony test in Belgium and the Netherlands. Facts, Views & Vision in ObGyn, 6, 7–12. PMC4086005 25009720

[mgg31256-bib-0047] Yang, H. C. , Wang, P. L. , Lin, C. W. , Chen, C. H. , & Chen, C. H. (2012). Integrative analysis of single nucleotide polymorphisms and gene expression efficiently distinguishes samples from closely related ethnic populations. BMC Genomics, 13, 346. 22839760 10.1186/1471-2164-13-346 PMC3453505

[mgg31256-bib-0048] Zhang, H. , Gao, Y. , Jiang, F. , Fu, M. , Yuan, Y. , Guo, Y. , … Wang, W. (2015). Non‐invasive prenatal testing for trisomies 21, 18 and 13: Clinical experience from 146,958 pregnancies. Ultrasound in Obstetrics and Gynecology, 45, 530–538. 10.1002/uog.14792 25598039

[mgg31256-bib-0049] Zhou, N. , & Wang, L. (2007). Effective selection of informative SNPs and classification on the HapMap genotype data. BMC Bioinformatics, 8, 484. 18093342 10.1186/1471-2105-8-484 PMC2245981

[mgg31256-bib-0050] Zimmermann, B. , Hill, M. , Gemelos, G. , Demko, Z. , Banjevic, M. , Baner, J. , … Rabinowitz, M. (2012). Noninvasive prenatal aneuploidy testing of chromosomes 13, 18, 21, X, and Y, using targeted sequencing of polymorphic loci. Prenatal Diagnosis, 32, 1233–1241. 10.1002/pd.3993 23108718 PMC3548605

